# The impact of universal induction therapy on early hospital readmission of kidney transplant recipients

**DOI:** 10.1590/2175-8239-JBN-2022-0042en

**Published:** 2022-11-11

**Authors:** Melissa Gaspar Tavares, Marina Pontello Cristelli, Julia Taddeo, Helio Tedesco Silva, Jose Medina Pestana

**Affiliations:** 1 Universidade Federal de São Paulo, Hospital do Rim e Hipertensão, Departamento de Nefrologia, São Paulo, SP, Brazil.

**Keywords:** Induction Therapy, Early Hospital Readmission, Kidney Transplantation, Mortality, Terapia de Indução, Readmissão Hospitalar Precoce, Transplante Renal, Mortalidade

## Abstract

**Background:**

Early hospital readmission (EHR) is associated with worse outcomes. The use of anti-thymocyte globulin (rATG) induction therapy is associated with increased efficacy in preventing acute rejection, although safety concerns still exist.

**Methods:**

This retrospective single-center study compared the incidence, causes of EHR, and one-year clinical outcomes of patients receiving a kidney transplant between August 18, 2011 and December 31, 2012 (old era), in which only high-risk patients received 5 mg/kg rATG, with those transplanted between August 18, 2014 and December 31, 2015 (new era), in which all patients received a single 3 mg/kg dose of rATG.

**Results:**

There were 788 patients from the Old Era and 800 from the New Era. The EHR incidence in the old era patients was 26.4% and in the new era patients, 22.5% (p = 0.071). The main cause of EHR in both eras was infection (67% vs. 68%). The incidence of acute rejection episodes was lower (22.7% vs 3.5%, p < 0.001) and the one-year patient survival was higher (95.6% vs. 98.1%, vs. p = 0.004) in new era patients.

**Conclusion:**

The universal use of 3 mg/kg rATG single-dose induction therapy in the new era was associated with a trend towards reduced EHR and a reduction in the incidence of acute rejection and mortality.

## Resumo

**Histórico:**

A Readmissão Hospitalar Precoce (RHP) está associada a piores desfechos. O uso de terapia de indução com globulina antitimócito (rATG, por sua sigla em inglês) está associado ao aumento da eficácia na prevenção de rejeição aguda, embora ainda existam preocupações quanto à segurança.

**Métodos:**

Este estudo retrospectivo de centro único comparou a incidência, as causas da RHP e os desfechos clínicos de um ano de pacientes que receberam transplante renal entre 18 de Agosto de 2011 e 31 de Dezembro de 2012 (Antiga Era), em que apenas pacientes de alto risco receberam 5 mg/kg de rATG, com aqueles transplantados entre 18 de Agosto de 2014 e 31 de Dezembro de 2015 (Nova Era), em que todos os pacientes receberam uma única dose de 3 mg/kg de rATG.

**Resultados:**

Houve 788 pacientes da Antiga Era e 800 da Nova Era. A incidência de RHP nos pacientes da antiga era foi de 26,4% e nos pacientes da nova era, 22,5% (p = 0,071). A principal causa de RHP em ambas as eras foi infecção (67% vs. 68%). A incidência de episódios de rejeição aguda foi menor (22,7% vs. 3,5%; p < 0,001) e a sobrevida do paciente em um ano foi maior (95,6% vs. 98,1%; vs. p = 0,004) em pacientes da nova era.

**Conclusão:**

O uso universal de terapia de indução de 3 mg/kg de rATG em dose única na nova era foi associado a uma tendência à redução da RHP e a uma redução na incidência de rejeição aguda e mortalidade.

## Introduction

Early hospital readmission (EHR) is a well-accepted measure of hospital quality. In the general population, EHR is also associated with increased morbidity and mortality^1,2^. Recent studies have shown that EHR is also a predictor of morbidity and mortality among kidney transplant recipients, increasing the risk of subsequent hospitalizations, graft loss, and death during the first year^3,4^.

In a previous analysis of a cohort of recipients of 1175 kidney transplants between January 2011 and December 2012, the incidence of EHR was 26.6%. Independent risk factors associated with EHR were recipient age, negative CMV serology, r-ATG induction therapy, treatment for acute rejection during index hospitalization and length of index hospital stay. The main cause of readmission was cytomegalovirus infection. Furthermore, EHR was associated with lower patient and graft survivals during the first year after transplantation^5^.

A recent single-center prospective randomized study demonstrated the efficacy and safety of the use of a single 3 mg/kg dose of rATG in kidney transplant recipients^6^. Since August 18, 2014, this strategy has been used in all patients at our institution, except recipients of kidneys from HLA identical living donors. Thus, the objective of this analysis was to compare the rate of EHR before and after the change in the use of induction therapy and its impact on short-term clinical outcomes.

## Methods

This was a single-center, retrospective, sequential cohort study that compared the incidence of EHR and one-year clinical outcomes of all adult patients receiving a kidney transplant between August 18, 2011 and December 31, 2012 (Old Era) and between August 18, 2014 to December 31, 2015 (New Era). The “New Era” period started immediately after the change in protocol, enabling a direct comparison with the previous era. The previous era was named “Old Era” so that it could be easily distinguished from the New Era throughout the study. All patients from both eras had a one-year follow-up. For this analysis, we excluded patients previously transplanted with other organs and patients with retransplants. We also excluded patients who died or lost the graft during the index hospital admission, patients participating in any clinical trial, and patients who were transferred from the center before one year of transplantation.

### Clinical Outcomes

The primary objective of this analysis was to compare the prevalence and causes of EHR between the two eras. The secondary objective was to compare the prevalence of treated acute rejection, patient- and death-censored graft survival, causes of death and graft loss, and kidney function within the first year after transplantation.

Early hospital readmission was defined as any hospitalization that occurred within 30 days after discharge from hospitalization for kidney transplantation. Causes of early hospital readmission were adjudicated and categorized as surgical complication requiring re-intervention, infections, cardiovascular events, metabolic disturbances (including electrolyte, anemia, and glycemic disorders), renal artery stenosis and kidney disease recurrence. Delayed graft function (DGF) was defined as the need for dialysis within the first week after transplantation. Expanded criteria donors (ECD) are those aged 60 years or over or donors aged 50–59 years with at least two out of three additional risk factors: stroke, history of high blood pressure, and serum creatinine above 1.5 mg/dL.

### Induction Therapy Strategy

In the Old Era, only patients with panel reactive antibodies (PRA) higher than 50% and recipients of kidneys from ECD received induction with rabbit antithymocyte globulin (rATG), with up to 6 mg/kg cumulative dose. Patients with PRA less than 50% receiving kidneys from living donors or standard deceased donors did not receive induction or received basiliximab. In the New Era, most of the patients received induction therapy with a single 3 m/kg dose of rATG. In both eras, patients who received kidneys from HLA identical living donors did not receive induction therapy.

Maintenance therapy, in both eras, consisted of tacrolimus or ciclosporine associated with azathioprine, mycophenolate, sirolimus, or everolimus, in addition to prednisone, depending on the perceived immunological risk. All patients received 1 g of methylprednisolone before the renal graft anastomosis during surgery and 30 mg of prednisone tapered to 5 mg/day between day 30 to day 45.

### Hospital Discharge Criteria

Patients from both protocols were discharged only after removal of all catheters and after recovery from delayed graft function (dialysis-free). None of the patients received pharmacological prophylaxis for cytomegalovirus infection. The use of universal pharmacological prophylaxis is associated with significant incidence of adverse events, primarily bone marrow toxicities, high cost, and lack of reimbursement. Instead, patients received preemptive therapy using pp65 antigenemia test for the first 3 months after transplantation. Asymptomatic patients with more than 10 positive cells per 200.000 leucocytes or symptomatic patients with any number of positive cells were treated with intravenous ganciclovir for at least 14 days or until viral clearance. Patients also received a 5-day course of albendazole for parasitic infection prophylaxis and trimetoprim sulfametoxazol for the prophylaxis of urinary tract infection and pneumocystis jirovecii pneumonia indefinitely.

### Statistical Analysis

Continuous variables were expressed as mean and standard deviation or as median and interquartile range, as needed. Categorical variables were summarized as frequency and percentage. Differences between groups were compared using the Student’s t-test or the chi-square test. Survival curves were obtained using the Kaplan-Meier method and comparisons were performed using the log-rank test (Mantel-Cox). Risk factors for EHR, acute rejection, death, graft loss, and reduce kidney function at 12 months were selected from the available literature. Variables with initial statistical significance in univariate analysis (p < 0.05) were inserted in the multivariable logistic regression analysis and the results presented as hazard ratio (HR) and 95% confidence interval (95% CI). Kidney function (estimated glomerular filtration rate, eGFR) was calculated using the CKD EPI formula^7^ and compared at one year using the imputation of the last observed carried forward value for patients who died or were lost to follow up. For patients with graft loss, the GFR was zero. Statistical analyses were performed using the SPSS v. 18.0 (SPSS Inc., Chicago, IL, USA).

## Results

### Population

**Figure 1.**
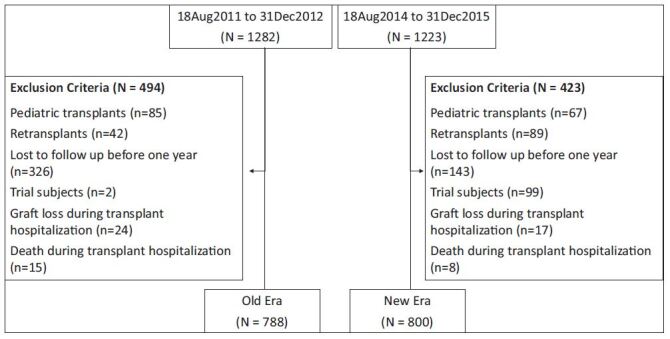
Population.

There were 1282 kidney transplants in the Old Era and 1223 in the New Era. of whom 1588 patients were eligible, 788 from the Old Era and 800 from the New Era (Figure 1). Patients from the New Era were older and had a higher proportion of history of smoking and alcohol abuse, hypertension, and diabetes mellitus (p < 0.001). The majority of patients were nonsensitized and received a high proportion of zero HLA-DR mismatch (69.5%), although a lower proportion of patients from the New Era had PRA Class II > zero and a slightly higher proportion received a zero HLA-B mismatch kidney compared to patients from the Old Era. As expected, a higher proportion of patients from the New Era received induction therapy, with a small imbalance in the use of azathioprine, mycophenolate, and mTOR inhibitors. In the New Era, 34 patients (4.3%) did not receive induction therapy because they received kidneys from HLA-identical living donors. There was a trend towards higher incidence of DGF but significantly lower incidence of treated acute rejection during hospital stay in the New Era group. The overall incidence of complications was lower in the New Era patients, primarily driven by lower incidence of infectious complications. Finally, the length of hospital stay was shorter in the New Era group(Table 1).

**Table 1.** Demographic characteristics, immunosuppression, and transplant hospital events

Parameter	Total	Old Era	New Era	p
	(n = 1588)	(n = 788)	(n = 800)	
**Recipient**				
Age, years*	46 (35–56)	45 (35–55)	47 (36–56)	0.040
Male n (%)	1001 (63)	491 (63.7)	510 (62.3)	0.552
Etiology of Chronic Kidney Decease, n (%)				<0.001
Glomerulonephritis	228 (14.4)	116 (14.7)	112 (14.0)	
Diabetes mellitus	224 (14.1)	82 (10.4)	142 (17.8)	
Hypertension	163 (10.3)	93 (11.8)	70 (8.8)	
Unknown	711 (44.8)	352 (44.7)	359 (44.9)	
Others	262 (16.5)	145 (18.4)	117 (14.6)	
Dialysis, n (%)	1536 (96.7)	763 (96.8)	773 (96.6)	0.821
Time on dialysis, years*	2.72 (1.33–4.93)	2.92 (1.17–5.00)	2.61 (1.40–4.62)	0.078
Virology, n (%)				
CMV IgG negative	96 (6.1)	49 (6.3)	47 (5.9)	0.720
HBV IgG positive	6 (0.4)	4 (0.5)	2 (0.3)	0.448
HCV IgG positive	16 (1.0)	9 (1.1)	7 (0.9)	0.591
HIV IgG positive	9 (0.6)	4 (0.5)	5 (0.6)	0.758
Comorbidities, n (%)				
Prior smoking	415 (26.1)	162 (20.6)	253 (31.6)	<0.001
Current smoking	89 (5.6)	48 (6.1)	41 (5.1)	0.403
Diabetes mellitus	241 (15.2)	90 (11.4)	151 (18.9)	<0.001
Hypertension	1320 (83.1)	614 (77.9)	706 (88.3)	<0.001
Congestive heart failure	39 (2.5)	23 (2.9)	16 (2.0)	0.237
Peripheral arterial disease	4 (0.3)	3 (0.4)	1 (0.1)	0.371
Chronic obstructive pulmonary disease	3 (0.2)	2 (0.3)	1 (0.1)	0.622
Coronary disease	77 (4.8)	34 (4.3)	43 (5.4)	0.325
Prior alcohol abuse	98 (6.2)	13 (1.6)	85 (10.6)	<0.001
Prior tuberculosis	12 (0.8)	9 (1.1)	3 (0.4)	0.078
Prior cancer	7 (0.4)	3 (0.4)	4 (0.5)	0.720
PRA Class I > zero, n (%)	413 (26.0)	212 (26.9)	201 (25.2)	0.436
PRA Class II > zero, n (%)	182 (11.5)	105 (13.3)	77 (9.7)	0.022
HLA zero mismatches, n (%)				
A	316 (20.0)	146 (18.7)	170 (21.3)	0.168
B	324 (20.5)	140 (17.9)	184 (23.1)	<0.001
DR	1098 (69.5)	530 (67.8)	568 (71.4)	0.302
**Donor**				
Age, years*	46 (36;55)	46 (37;55)	46 (36;54)	1.000
Type				
Living, n (%)	409 (25.8)	213 (27)	196 (24.5)	0.320
Deceased, n (%)				0.348
Standard, n (%)	750 (47.3)	358 (45.5)	392 (49.1)	
Expanded, n (%)	425 (26.8)	216 (27.4)	209 (26.2)	
Cold ischemia time (deceased donor), hours*	22 (19–27)	22 (19–26)	23 (19–28)	0.061
**Immunosuppression**				
Induction therapy, n (%)				<0.001
None	329 (20.7)	295 (37.4)	34 (4.3)	
rATG	1058 (66.6)	292 (37.1)	766 (95.8)	
Basiliximab	201 (12.7)	201 (25.5)	0 (0)	
Maintenance, n (%)				<0.001
Calcineurin inhibitor + Azathioprine	810 (51.1)	337 (42.8)	473 (59.4)	
Calcineurin inhibitor + Mycophenolate	593 (37.4)	303 (38.5)	290 (36.4)	
Calcineurin inhibitor + mTOR inhibitor	165 (10.4)	132 (16.8)	33 (4.1)	
mTOR inhibitor + Mycophenolate	16 (1.0)	16 (2.0)	0 (0)	
**Transplant hospitalization**				
Delayed graft function, n (%)	682 (42.9)	320 (40.6)	362 (45.3)	0.062
Treated acute rejection, n (%)	207 (13.0)	179 (22.7)	28 (3.5)	<0.001
Complications, n (%)	239 (15.1)	143 (18.1)	96 (12.0)	0.033
Surgical	89 (5.6)	45 (5.71)	44 (5.5)	
Infectious	123 (7.7)	85 (10.8)	38 (4.7)	
Cardiovascular	23 (1.4)	12 (1.5)	11 (1.4)	
Metabolic	3 (0.18)	1 (0.12)	2 (0.2)	
Recurrence of glomerulonephritis	1 (0.06)	0 (0)	1 (0.1)	
Length of stay, days*	10 (7–17)	9 (7–15)	8 (6–13)	0.025

*Median (interquartile range); CMV: cytomegalovirus; HBV: Hepatitis B virus; HCV: Hepatitis C virus; HIV: Human Immunodeficiency Virus; PRA: Panel Reactive Antibody; mTOR: mammalian target of rapamycin.

### Early Hospital Readmission

**Figure 2.**
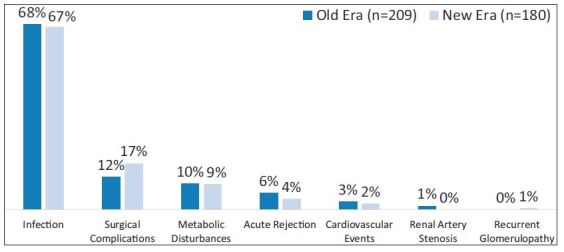
Causes of EHR.

**Figure 3.**
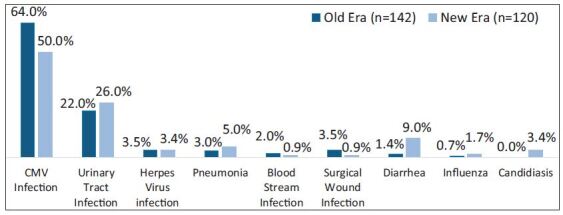
Specific infectious causes of EHR.

There was a trend towards a reduction in the incidence of EHR in the New Era patients compared with Old patients (22.5% vs. 26.4%; p = 0.071). In both eras, the main cause of readmission was infectious (67% vs. 68%) followed by surgical (17% vs. 12%) and metabolic complications (9.4% vs. 9.6%) (Figure 2). In both eras, approximately 6% of recipients had negative CMV serology (Old Era 6.3% vs 5.9% in the New Era, p = 0.720, Table 1). Yet, compared to the Old Era, there was a reduction in EHR due to CMV infection (64% vs. 50%, p = 0.006) in the New Era. There was an increase of EHR associated with diarrhea (1.4% vs. 9%, p = 0.013) in the New Era (Figure 3). There was no difference in time from index discharge to readmission (13 [IQR 8–22] vs. 12 [IQR 6–20] days, p = 0.678) or in length of hospital stay during EHR (15 [IQR 8–25] vs. 13 [IQR 5–21] days, p = 0.157). The independent risk factors associated with EHR were older recipient, CMV-negative recipient, and longer DGF period (Table 2).

**Table 2.** Risk factors associated with early hospital readmission

Parameter	Univariate analysis		Multivariate analysis	
	HR (95% CI)	p value	HR (95% CI)	p value
Recipient age >46 years	1.89 (1.5–2.38)	<0.001	1.66 (1.28–2.15)	**<0.001**
Time on dialysis >2.7 years	1.46 (1.16–1.84)	0.001	1.15 (0.86–1.44)	0.40
Diabetes mellitus, yes	1.23 (0.88–1.73)	0.219	0.78 (0.50–1.21)	0.28
CMV IgG, negative	2.14 (1.39–3.27)	<0.001	2.35 (1.48–3.73)	**<0.001**
PRA Class I >0	1.43 (1.11–1.84)	0.005	1.2 (0.89–1.6)	0.22
PRA Class II >0	1.53 (1.09–2.14)	0.013	1.33 (0.91–1.94)	0.141
Prior tuberculosis	1.16 (0.90–1.50)	0.253	–	
Prior alcoholism	0.83 (0.50–1.37)	0.475	–	
Donor age >46 years	1.60 (1.26–2.01)	<0.001	1.19 (0.87–1.64)	0.26
Donor type			–	
Living	Reference			
Deceased standard	1.92 (1.39–2.65)	<0.001	1.10 (0.72–1.67)	0.64
Deceased expanded	3.04 (2.16–4.29)	<0.001	1.58 (0.98–2.52)	0.055
Cold ischemia time >22 hours	1.57 (1.25–1.98)	<0.001	1.23 (0.94–1.61)	0.12
Delayed graft function, yes	1.84 (1.47–2.32)	<0.001	1.32 (0.95–1.82)	0.091
Delayed graft function >9 days	0.72 (0.52–0.98)	0.038	0.64 (0.46–0.90)	**0.010**
Transplant complication	1.70 (1.32–2.20)	<0.001	1.36 (0.92–2.02)	0.12
Acute rejection during transplant hospital stay	1.5 (1.09–2.06)	0.013	1.16 (0.73–1.85)	0.52
Length of transplant hospital stay >10 days	1.83 (1.45–2.31)	<0.001	1.14 (0.81–1.61)	0.42
Era				
Old	Reference		Reference	
New	0.81 (0.64–1.01)	0.071	0.81 (0.62–1.05)	0.11

### Treatment Failure

**Figure 4.**
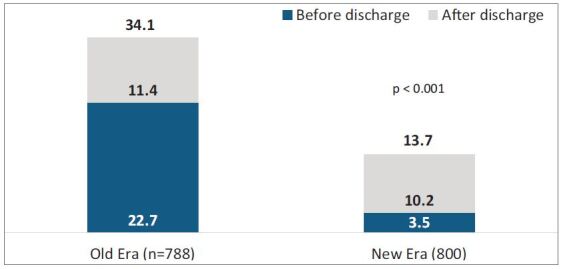
Total acute rejection.

The overall incidence of treated acute rejection episodes decreased from 34.1% in the Old Era to 13.3% in the New Era cohorts (p < 0.001, Figure 4). This significant reduction was associated with lower rates of treated acute rejection during index hospital stay (22.7 vs. 3.5%, p < 0.001). There was a significant reduction in mortality in the New Era (1.87 vs. 4.44%, p = 0.003), primarily due to reduction of infection-associated deaths. There were no differences in the incidence and causes of graft loss (Table 3).

**Table 3.** Treatment failure

Parameters	Old Era (n = 788)	New Era (n = 800)	p
Treated acute rejection, n (%)	269 (34.1)	110 (13.8)	<0.001
Total graft loss, n (%)	46 (5.83)	28 (3.5)	0.027
Death, n (%)	35 (4.44)	15 (1.87)	0.003
Infectious	26 (74.2)	8 (53.3)	
Cardiovascular	1 (2.8)	3 (20)	
Unknown	6 (17.1)	2 (13.3)	
Surgical Complication	2 (5.7)	0	
Cancer	0	1 (6.67)	
Cerebrovascular	0	1 (6.67)	
Time to death, days, median (IQR)	115 (71–217)	160 (32–236)	0.306
Graft Loss, n (%)	11 (1.40)	13 (1.62)	0.708
Primary Non function	0	2 (15.4)	
Venous thrombosis	0	1 (7.7)	
Non-Immune IFTA	2 (18)	2 (15.4)	
Pyelonephritis	1 (9)	0	
Immune IFTA	2 (18)	3 (23)	
Discontinuation of Immunosuppression	4 (36)	0	
Acute Rejection	1 (9)	4 (30.7)	
Uremic Hemolytic Syndrome	1 (9)	0	
Thrombotic Microangiopathy	0	1 (7.7)	
Time to graft loss, days, median (IQR)	135 (91–227)	131 (35.7–227)	0.811

**Figure 5.**
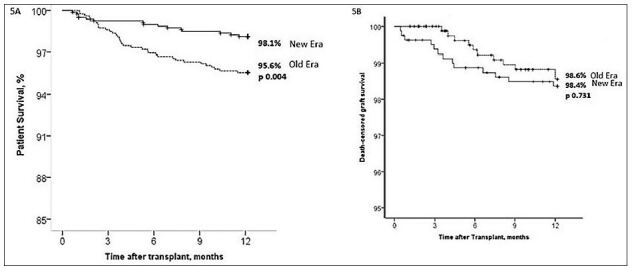
Patient survival. (B) Death – censored graft survival.

The risk factors associated with incidence of treated acute rejection were younger recipient age, delayed graft function, and need for EHR. Receiving a kidney transplant in the New Era was associated with 70% reduction in the risk of acute rejection (Table 4). Patient survival was higher in the New Era (98.1% vs. 95.6%, p = 0.004, Figure 5A). The independent risk factors associated with death within one year were older donor age and need for EHR. Patients receiving a kidney transplant in the New Era had a 63% lower risk of death (Table 5). There was no difference in death-censored graft survival between the Old and New Eras (98.4% vs. 98.6%, p = 0.731, Figure 5B, respectively). The independent risk factors for graft loss within one year were: donor age >46 years – median [2.25 (1.15–4.40) p = 0.017], cold ischemia time greater than the median 22 hours [1.76 (1.01–3.06) p = 0.044] and EHR [2.92 (1.76–4.85) p < 0.001] (Table S1).

**Table 4.** Risk factors associated with incidence of treated acute rejection

Parameter	Univariate analysis		Multivariate analysis	
	HR (95% CI)	p value	HR (95% CI)	p value
Recipient age >46 years	0.66 (0.52–0.83)	0.001	0.65 (0.50–0.84)	0.001
Time on dialysis >2.7 years	1.14 (0.90–1.43)	0.26	–	
Diabetes mellitus, yes	0.73 (0.43–1.24)	0.25	–	
CMV IgG, negative	1.19 (0.63–2.24)	0.58	–	
PRA Class I >0	1.22 (0.86–1.73)	0.26	–	
PRA Class II >0	0.98 (0.68–1.41)	0.92	–	
Zero HLA A mm	1.33 (0.91–1.94)	0.138	–	
Zero HLA B mm	1.27 (0.87–1.87)	0.21	–	
Zero HLA DR mm	1.07 (0.75–1.53)	0.67	–	
Donor age >46 years	1.01 (0.80–1.27)	0.92	–	
Donor type			–	
Living	Reference			
Deceased standard	0.83 (0.55–1.25)	0.38	–	
Deceased expanded	1.13 (0.74–1.73)	0.56	–	
Cold ischemia time >22 hours	0.83 (0.65–1.06)	0.15	–	
Delayed graft function, yes	1.48 (1.17–1.87)	0.001	1.63 (1.27–2.10)	<0.001
Delayed graft function >9 days	0.95 (0.69–1.32)	0.78	–	
Transplant complication	0.88 (0.54–1.45)	0.64	–	
Length of transplant hospital stay >10 days	0.82 (0.56–1.18)	0.29	–	
EHR, yes	2.04 (1.45–2.87)	<0.001	1.83 (1.39–2.40)	<0.001
Era				
Old	Reference		Reference	
New	0.68 (0.49–0.94)	0.021	0.30 (0.24–0.98)	<0.001

**Table 5.** Risk factors associated with death within one year after kidney transplantation

Parameter	Univariate analysis		Multivariate analysis	
	HR (95% CI)	p value	HR (95% CI)	p value
Recipient age >46 years	2.50 (1.35–4.62)	0.003	1.62 (0.81–3.26)	0.169
Time on dialysis >2.7 years	1.65 (0.92–2.95)	0.088	–	
Diabetes mellitus, yes	2.15 (1.08–4.28)	0.029	2.13 (0.96–4.72)	0.062
Coronary disease, yes	1.45 (0.56–3.74)	0.43	–	
Current smoking, yes	1.92 (0.74–4.97)	0.17	–	
Prior smoking, yes	1.1 (0.59–2.06)	0.76	–	
Prior tuberculosis, yes	6.36 (1.35–29.8)	0.019	4.03 (0.72–22.50)	0.111
Alcoholism, yes	0.97 (0.29–3.17)	0.95	–	
Prior cancer, yes	5.21 (0.61–44.1)	0.13	–	
CMV IgG negative, yes	1.0 (0.30–3.29)	0.992	–	
Positive serology for HCV, yes	4.53 (1.01–20.48)	0.050	3.60 (0.66–19.50)	0.137
PRA Class I > zero, yes	1.83 (1.02–3.30)	0.042	1.76 (0.92–3.33)	0.083
PRA Class II > zero, yes	1.52 (0.70–3.31)	0.283	–	
Zero HLA mismatches				
A	1.27 (0.65–2.46)	0.47	–	
B	0.42 (0.16–1.07)	0.069	–	
DR	0.84 (0.46–1.53)	0.57	–	
Cold ischemia time >22 hours	1.72 (0.98–3.02)	0.059	–	
Donor age >46 years	4.06 (2.0–8.24)	<0.001	2.66 (1.12–6.34)	0.027
Donor type				
Living	Reference		Reference	
Standard deceased donor	1.33 (0.55–3.24)	0.52	0.82 (0.27–2.48)	0.73
Extended deceased donor	3.59 (1.53–8.41)	0.003	1.08 (0.36–3.24)	0.88
Delayed graft function, yes	1.87 (1.05–3.31)	0.031	1.25 (0.57–2.73)	0.57
Delayed graft function >9 days	0.59 (0.25–1.40)	0.233	–	
Length of transplant hospital stay >10 days	1.99 (1.13–3.50)	0.017	1.33 (0.66–2.70)	0.41
Transplant complications, yes	1.69 (0.93–3.07)	0.084		
Early hospital readmission, yes	4.16 (2.35–7.37)	<0.001	3.14 (1.68–5.85)	<0.001
Treated acute rejection, yes	0.79 (0.39–1.59)	0.515	–	
Era				
Old	Reference		Reference	
New	0.411 (0.22–0.76)	0.004	0.37 (0.19–0.73)	0.004

### Kidney Function

The incidence of DGF tended to be higher in the New Era (Old Era 40.6% vs New Era 45.2%, p = 0.062), but the duration was shorter [Old Era – 10 days, median (IQR) (6–13) vs New Era – 8 days, median (IQR) (5–11) p < 0.001]. There was no difference in one-year median eGFR between the eras [Old Era – 55.26 mL/min/1.73m^2^, median (IQR) (40.7–71.8); New Era – 54.18 mL/min/1.73m^2^, median (IQR) (40.7–68.9) p = 0.266] (Table S2). In this cohort, there were no independent risk factors associated with reduced kidney function at one year (Table S3).

## Discussion

This retrospective analysis suggests that the use of a universal single 3 mg/kg rATG dose as induction therapy in the New Era was associated with a trend towards reduction in EHR and ultimately higher one-year patient survival, despite the higher age and number of comorbidities, including diabetes mellitus, all of which are known risk factors associated with EHR^3,4,5,6,7,8,9,10^.

In the Old Era, 37.1% of the patients received rATG and 25.5% basiliximab, while 95.6% of the patients received rATG in the New Era. Interestingly, despite the trend of increased incidence of DGF in the New Era, the prevalence of acute rejection and infectious complications was reduced, perhaps leading to a shorter length of stay.

Independent risk factors associated with EHR were recipient with older age and CMV IgG-negative, and prolonged duration of DGF, but the Era was not. In our previous analysis, rATG induction was an independent risk factor for EHR, indicating that the magnitude of the dose used previously, 5 mg/kg, is perhaps involved^5^. The primary cause of EHR was infection followed by surgical complications. CMV infection accounted for most infections associated with her, as preemptive therapy was used in all patients in both Eras^11,12^. Remarkably, readmission associated with CMV infection was lower in the New Era, suggesting that treatment for early acute rejection is a predominant risk factor compared to universal reduced dose of rATG as induction therapy. Furthermore, the reciprocal causal interaction between acute rejection and CMV infection is known to lead to increased morbidity and mortality in the first year after transplantation^12,13,14,15^. In fact, use of induction therapy has been associated with a reduced risk of EHR, possibly through the reduction of the incidence of early acute rejection^3,4^.

There were differences in clinical outcomes between the eras. At one year, patients who received a kidney transplant in the New Era showed a lower incidence of treated acute rejection but had similar rates of kidney graft function and graft loss. Risk factors associated with increased risk of acute rejection were younger age, DGF, and receiving a kidney transplant in the Old Era, while cold ischemia time, older donor age, and receiving a kidney transplant in the Old Era were associated with higher mortality. Notably, EHR was associated with increased risk of acute rejection, graft loss, and death. Previous studies have shown that EHR is associated with higher mortality and graft loss in the first year of transplantation^3,4,8,16^. There is certainly an interaction between EHR and transplant era. While EHR is a proxy mostly associated with unfavorable demographic characteristics, universal induction therapy in the New Era was associated with lower incidence of treatment for acute rejection and perhaps lower infectious complications inherent to its treatment, especially in frail patients^17^.

The retrospective analysis has intrinsic limitations, including potential population selection bias, specific regional demographic characteristics, and clinical practice strategies that evolved over time. Therefore, interpretation and extrapolation of these data should be made with caution. Both eras were followed-up for one year after transplantation, so we did not have additional information about the change in the institutional protocol after this period and in the long term.

In summary, EHR is a relevant medical quality measure that is associated with worse clinical outcomes after kidney transplantation. The change of the induction therapy strategy introduced in the New Era was associated with a trend towards reduced EHR and significant reduction in the incidence of treated acute rejection and mortality.

## Supplementary Material

Table S1 – Risk factors associated with graft loss in one year after kidney transplantation.

Table S2 – Kidney function.

Table S3 – Risk factors associated with low one-year estimated glomerular filtration rate
