# Supplementary material for: The impact of universal induction therapy on early hospital readmission of kidney transplant recipients
Source: J Bras Nefrol. 2022 Nov 11;45(2):218–28. doi: 10.1590/2175-8239-JBN-2022-0042en (PMC10627129; doi:10.1590/2175-8239-JBN-2022-0042en)
Supplement: Supplementary file 1 [file 2175-8239-jbn-45-2-e20220042-s1.pdf]

Braz. J. Nephrol. (J. Bras. Nefrol.)

https://doi.org/10.1590/2175-8239-JBN-2022-0042en

Supplementary Material to "The impact of universal induction therapy on early hospital readmission of kidney transplant recipients"

Table S1 - Risk Factors associated with graft loss within one year after kidney transplantation.

Parameters	Univariate analysis		Multivariate analysis	
	HR (95% CI)	p value	HR (95% CI)	p value
Recipient age > 46 years	1.39 (0.87-2.22)	0.169		
Time on dialysis > 2.7 years	1.45 (0.92-2.40)	0.098		
Diabetes mellitus	1.46 (0.77-2.76)	0.243		
CMV IgG negative, yes	1.16 (0.45-2.94)	0.756		
Previous tuberculosis, yes	4.17 (0.90-16.41)	0.068		
PRA Class I > zero	1.51 (0.91-2.49)	0.756		
PRA Class II > zero	1.39 (0.71-2.69)	0.327		
Zero HLA mismatches				
A	1.10 (0.62-1.95)	0.723		
B	0.59 (0.3-1.16)	0.130		
DR	0.70 (0.43-1.14)	0.157		
Cold ischemia time, > 22 hours	2.18 (1.36-3.5)	0.001	1.76 (1.01-3.06)	0.044
Donor age > 46 years	2.97 (1.74-5.09)	<0.001	2.25 (1.15-4.40)	0.017
Donor type				
Living	reference		reference	
Deceased standard	1.60 (0.77-3.33)	0.201	0.93 (0.37-2.34)	0.880
Deceased expanded	3.47 (1.69-7.13)	0.001	1.08 (0.41-2.84)	0.876
Delayed graft function	2.56 (1.5- 4.17)	<0.001	1.45 (0.73-2.86)	0.285
Delayed graft function >9 days (median)	0.677 (0.34-1.33)	0.260		
Length of transplant hospital stay >10 days	2.69 (1.67-4.30)	<0.001	1.54 (0.78-3.02)	0.210
Transplant complications, yes	1.91 (1.17-3.12)	0.009	1.17 (0.60-2.28)	0.627
Early hospital readmission, yes	3.94 (2.45-6.32)	<0.001	2.92 (1.76-4.85)	<0.001
Total acute rejection, yes	1.67 (1.01-2.75)	0.042	1.25 (0.69-2.26)	0.455
Era				
Old	reference		reference	
New	0.58 (0.36-0.94)	0.029	0.66 (0.39 – 1.13)	0.133
