# Supplementary material for: The impact of universal induction therapy on early hospital readmission of kidney transplant recipients
Source: J Bras Nefrol. 2022 Nov 11;45(2):218–28. doi: 10.1590/2175-8239-JBN-2022-0042en (PMC10627129; doi:10.1590/2175-8239-JBN-2022-0042en)
Supplement: Supplementary file 2 [file 2175-8239-jbn-45-2-e20220042-s2.pdf]

Braz. J. Nephrol. (J. Bras. Nefrol.)

https://doi.org/10.1590/2175-8239-JBN-2022-0042en

Supplementary Material to "The impact of universal induction therapy on early hospital readmission of kidney transplant recipients"

Table S2 - Kidney function.

Parameters	Old Era (n=788)	New Era (n=800)	p
DGF, n (%)	320 (40.6)	362 (45.2)	0.062
DGF duration, days, median (IQR)	10 (6 - 13)	8 (5 - 11)	< 0.001
DGF > 9 days, n (%)	164 (20.8)	130 (16.3)	0.019
1-year eGFR, mL/min/1.73m^2, median (IQR)	55.26 (40.76 - 71.8)	54.18 (40.7-68.9)	0.266
1-year eGFR > 54 mL/min/1.73m^2, n (%)	402 (51)	383 (47.9)	0.211

eGFR: Estimated Glomerular Filtration Rate; DGF: Delayed Graft Function.
