# Supplementary material for: The impact of universal induction therapy on early hospital readmission of kidney transplant recipients
Source: J Bras Nefrol. 2022 Nov 11;45(2):218–28. doi: 10.1590/2175-8239-JBN-2022-0042en (PMC10627129; doi:10.1590/2175-8239-JBN-2022-0042en)
Supplement: Supplementary file 3 [file 2175-8239-jbn-45-2-e20220042-s3.pdf]

Braz. J. Nephrol. (J. Bras. Nefrol.)

https://doi.org/10.1590/2175-8239-JBN-2022-0042en

Supplementary Material to "The impact of universal induction therapy on early hospital readmission of kidney transplant recipientes"

Table S3 - Risk factors associated with low (< 54 mL/min/1.73m^2) one-year estimated glomerular filtration rate (eGFR).

**Parameters**	**Univariate analysis HR (95% CI)**	**p value**	**Multivariate analysis HR (95% CI)**	**p value**
Recipient age > 46 years	1.22 (1.00-1.49)	0.044	1.15 (0.94-1.40)	0.16
Time on dialysis > 2.7 years	0.96 (0.79-1.17)	0.72	-	
Diabetes mellitus, yes	1.01 (0.74-1.37)	0.94	-	
CMV IgG, negative	0.86 (0.57 – 1.30)	0.47	-	
PRA Class I > zero, yes	1.06 (0.84-1.32)	0.61	-	
PRA Class II > zero, yes	0.97 (0.71-1.33)	0.87	-	
Cold ischemia time > 22 hours	0.99 (0.81-1.22)	0.97	-	
Delayed graft function, yes	1.06 (0.87-1.3)	0.53	-	
Delayed graft function > 9 days	0.99 (0.76-1.27)	0.93	-	
Complications during transplant hospitalization	0.91 (0.72-1.15)	0.47	-	
Donor age > 46 years	1.23 (1.01-1.50)	0.039	1.20 (0.99-1.47)	0.064
Donor type				
Living	Reference			
Standard	0.91 (0.72-1.16)	0.47	-	
Expanded	1.14 (0.87-1.50)	0.32	-	
EHR, yes	0.99 (0.79-1.25)	0.98	-	
Treated Acute Rejection, yes	0.99 (0.78-1.24)	0.94	-	
Era				
Old	Reference			
New	1.13 (0.93-1.38)	0.21		

PRA: panel reactive antibodies; EHR: early hospital readmission.
